# Health Conditions and Long Working Hours in Europe: A Retrospective Study

**DOI:** 10.3390/ijerph191912325

**Published:** 2022-09-28

**Authors:** Darja Korošec, Dominika Vrbnjak, Gregor Štiglic

**Affiliations:** 1 Faculty of Health Sciences, University of Maribor, 2000 Maribor, Slovenia; 2 Faculty of Electrical Engineering and Computer Science, University of Maribor, 2000 Maribor, Slovenia; 3 Usher Institute, University of Edinburgh, Edinburgh EH8 9YL, UK

**Keywords:** working environment, workplace, working time, occupational health, employees’ health, health outcome, hypertension, cholesterol, diabetes, heart attack, employees, healthcare workers

## Abstract

Studies have shown that working conditions and employee health are related; therefore, this study aimed to determine the relationships between working long hours per week with health outcomes in different European countries. We analyzed data derived from the Survey of Health, Ageing, and Retirement in Europe. The sample included 12.099 participants aged ≥ 50 from 16 European countries. We analyzed self-reported working hours, health outcomes of blood cholesterol, heart attack, diabetes, and hypertension, as well as a longitudinal analysis of health outcomes among healthcare workers and workers in 13 other industries. Statistical analyses identified that men are working longer hours per week compared with women in all included countries, and different levels of increase in health conditions in different industries. We also observed a slower increase in the prevalence of health conditions for healthcare workers compared with workers in other industries, especially diabetes and hypertension. The largest increase in prevalence of observed health conditions was reported for cholesterol, which increased for 17.14% among healthcare workers and for 21.70% in other industries over the observed nine-year period. Although the data point to a potentially high level of awareness in the field of preventive health among healthcare workers, more preventive health activities should be included in workplaces to strengthen employees’ health.

## 1. Introduction

The workplace is where adults spend most of their time, preceded only by the home environment. During that time, they may experience different effects of working conditions on their health. According to the World Health Organization, more than two million people die from work-related injuries and diseases every year. The most common are noncommunicable diseases, which contribute 70%, injuries 22%, and infectious diseases 8% of the total disease burden of occupational risks [1]. The European Agency for Safety and Health at Work (EU-OSHA) estimates that 3.9% of the world gross domestic product (GDP) and 3.3% of the European GDP are spent on occupational diseases and injuries [2]. Over 200,000 people die each year from work-related illnesses in Europe. The leading cause of work-related deaths is cancer in 52%, followed by circulatory diseases (heart disease or stroke) in 24%, other causes in 22%, and injuries in 2% [3]. The incidence of the most common noncommunicable diseases (cardiovascular disease, cancer, COPD, and diabetes) increases with age in males and females. Their prevalence is the highest in the population over 75 years of age. Still, the common incidence of chronic diseases is already significant in people of working and retirement age in many European countries. About 52 million Europeans aged 55 to 74 years reported having a long-term illness or health problem [4]. In European countries, approximately one in four people of all ages has at least one long-standing illness. Hypertension is the most common, followed by diabetes mellitus, depressive disorders, asthma, and COPD [5].

Countries at the most risk for occupational death and disease are those with low and middle income. Moreover, some workplaces are at higher risk for work-related diseases and injuries, such as mining, construction, agriculture, and manufacturing. Work-related diseases are also connected to specific workplaces; for example, the risk of stress and musculoskeletal diseases is higher among office workers; overexertion and infections are more often among healthcare workers [1]. Epidemiological data point at a correlation between coronary heart disease and chronic stress. The risk of developing coronary heart disease is higher in employees who experience work-related stress [6]. Excessive workload, poor work–life balance, lack of involvement in making decisions that affect the worker, lack of role clarity, conflicting demands, lack of support from managements or colleagues, and working with difficult clients, patients, pupils, students, or customers impact worker’s mental and physical health [7].

Exposure to work-related stress is a significant feature for healthcare workers. Despite this, the prevalence of diabetes and hypertension is lower among healthcare workers in comparison to the general population. However, prevalence is increasing, despite the assumption that healthcare workers are opting for healthier lifestyles and better health outcomes due to greater health literacy, experiences with patients, and education [8]. In addition to specific stressors, such as emotionally demanding environments with patients and their families, healthcare workers work under an extensive workload; they work long night shifts and many working hours [9] because of the complexity of a sector that has to ensure services 24 h every day of the year [10]. In a cross-sectional study with a sample of 31,627 registered nurses in 12 European countries, it was found that 15% of nurses worked shifts of 12 hours or more [11] despite the European Working Time Directive, which sets limits of 48 h per week, including overtime. It provides a minimum daily rest period of 11 h [12].

In 2016, 479 million people, or 9% of the global population, were working at least 55 h/week [13]. In European countries, people worked 36.4 h on average per week in 2021. Working hours varied across the countries from 32.2 h in the Netherlands to 40.1 h in Greece [14]. Working 55 or more hours per week increases the risk of stroke by 35% [13]. The results of a European study show that, among analyzed employees in European countries, full-time schedules (35–40 h per week) were reported most frequently but part-time work is also on the rise in the analyzed period from the year 2005 to 2015 [15]. Connections were found between long working hours and health among men in countries with male breadwinner models in a European study. They found associations between working 51–60 h per week with work-related poor health status, stress, and psychological distress. The study included a sample of 9288 men and 6295 women aged 16–64 years working 30–60 h per week [16]. In a cross-sectional study based on data from the European Working Conditions Survey 2010, which included 13,518 men and 9381 women, they found associations between working long hours and poor health status and poor psychological wellbeing in countries with traditional family models by both sexes in liberal countries and among women in southern and continental European countries [17].

As mentioned, some studies already explored the correlations between long working hours and health conditions but not on the pan-European level. This study aimed to explore the connections between working long hours per week and self-perceived health status among different working professions, especially healthcare workers in different European countries. We focused on the older adult population, where chronic conditions are more prevalent compared with younger generations of the working population.

## 2. Materials and Methods

### 2.1. Database

Data presented here and used in our study were derived from the Survey of Health, Ageing and Retirement in Europe (SHARE) from the fourth wave, comprised of the data collected in the year 2010/2011, compared with the eighth wave collected in 2019/2020. The SHARE database is a multidisciplinary and multinational database of microdata on socioeconomic status, health and social, and family networks of participants aged 50 years and older in European countries. From 2004 to 2020, there have been eight waves of the study, which included 530,000 surveys with 140,000 participants from 28 European countries and Israel [18].

### 2.2. Sample

The SHARE database includes a random sample of participants who are 50 years old or older and have their regular domicile in the respective SHARE country. In addition to each original sample member in the SHARE database, their partner who lives at the same address as the respondent is interviewed if he agrees to participate, regardless of his age, which means that he may be under 50 years old. Excluded are those who are in prison; hospitalized or absent from the country for the duration of the field data collection; do not speak the official language; or have moved and the new address is unknown [19]. To allow segmentation by the business, industry, or services where the participants were currently employed, we used only data from participants where the industry sector information was available. The following 14 industries are represented in SHARE wave 4 data: 1. agriculture, hunting, forestry, and fishing; 2. mining and quarrying; 3. manufacturing; 4. electricity, gas, and water supply; 5. construction; 6. wholesale and retail trade, repair of motor vehicles, motorcycles, and personal and household goods; 7. hotels and restaurants, 8. transport, storage, and communication; 9. financial intermediation; 10. real estate, renting, and business activities; 11. public administration and defense; compulsory social security; 12. education; 13. health and social work; and 14. other community, social, and personal service activities. 

### 2.3. Variables

#### 2.3.1. Country Typology

The fourth wave in the SHARE database included 16 European countries, which we classified according to geographical position in four groups: *Western Europe* (Austria, Belgium, Germany, France, and Switzerland); *Eastern Europe* (Czech Republic, Estonia, Hungary, Slovenia, and Poland); *Southern Europe* (Spain, Italy, and Portugal); and *Northern Europe* (Denmark, Sweden, and The Netherlands).

#### 2.3.2. Health Assessment and Workload Characteristics

We included data on the health status of participants in the SHARE database. Participants were asked about the following diseases: *long-term illness; heart attack; high blood pressure; high blood cholesterol; stroke; diabetes* or *high blood sugar; chronic lung disease;* and *asthma*. Participants have identified whether they ever had the disease or currently have it, so possible answers were *ever diagnosed* and *currently have it*. The results section includes the data on the four most frequent health conditions. Another important piece of information on working conditions was working hours per week. Participants were asked: *“How many hours do you work per week?*”.

### 2.4. Data Analysis

In the data preprocessing step, we renamed the variables into more meaningful categories and merged the data from different waves. In case of missing data for key variables (e.g., industry or country), we removed participants from further analysis. For longitudinal analyses, only participants whose data for all waves of data between wave 4 (2011) and wave 8 (2019/2020) were available were included. We visualized the confidence intervals, which allow easier estimation of the variance and difference between the compared groups. Visualization of the longitudinal comparison of health condition prevalence was conducted based on self-reported cases up to the year of observation. For example, if participants reported diabetes in 2011 and 2013, we counted them as diabetes patients even if they did not report the diagnosis of diabetes in 2015. All statistical analyses were conducted using the R programing language [20,21].

## 3. Results

### 3.1. General Description of the Sample

**Figure 1:**
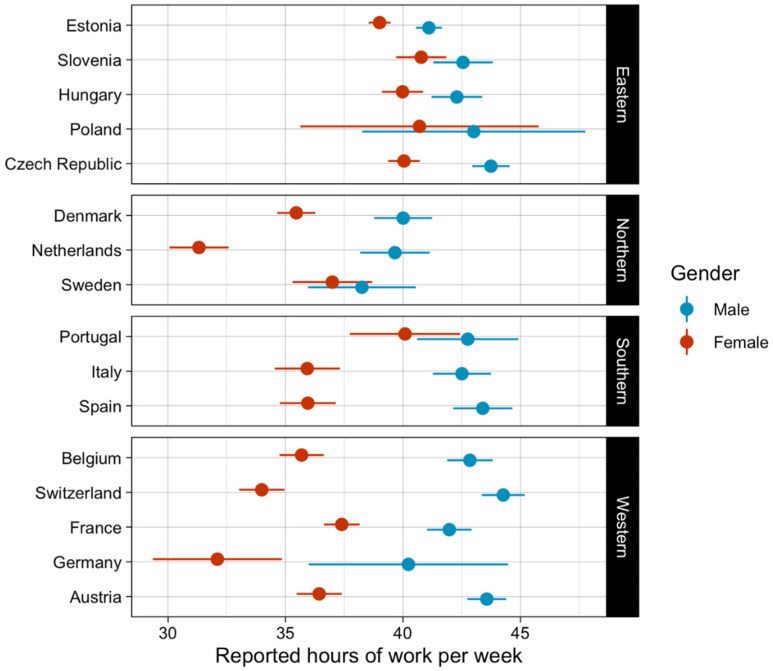
Hours of work per week by country and gender.

Out of 58,121 participants in wave 4 of the SHARE study, 12,099 fulfilled the eligibility criteria. Of all participants, 5598 (46%) were men, and 1485 (12%) worked in the health and social work institutions. Figure 1 presents reported working hours per week by country and gender. Participants from different countries were working from 30 to 45 h per week on average. Results show that men are working longer hours per week in comparison to women in all included countries. There were three countries where women worked less than 35 h per week: Netherlands, Switzerland, and Germany. Men in all countries, except Netherlands and Sweden, work 40 or more hours per week. Figure 1 also shows that participants in Eastern Europe work longer hours per week compared with Northern, Southern, and Western Europe, regardless of gender. 

### 3.2. Between-Country Differences in Health Conditions by Long Working Hours

**Figure 2:**
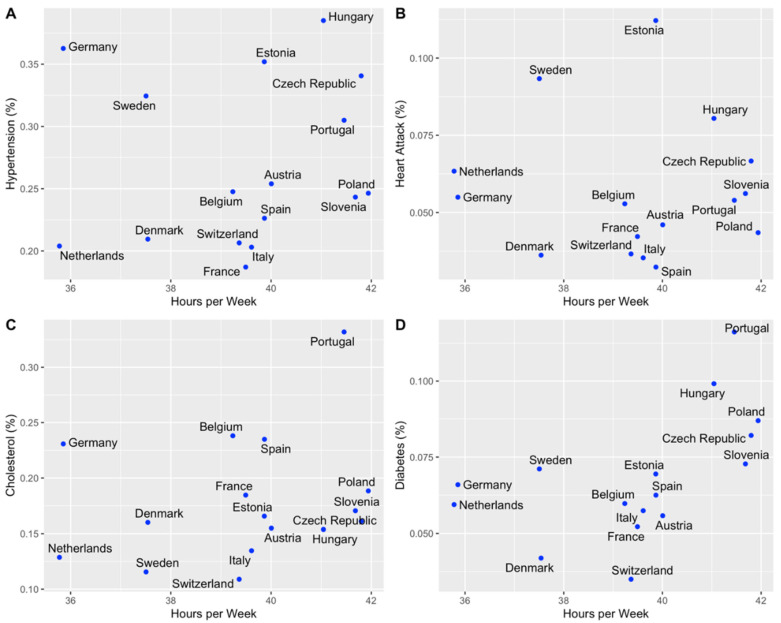
Association of reported health conditions and working hours by country. (**A**) represents the prevalence of hypertension in included countries by working hours per week. (**B**) represents the prevalence of heart attack in included countries by working hours per week. (**C**) represents the prevalence of cholesterol in included countries by working hours per week. (**D**) represents the prevalence of diabetes in included countries by working hours per week.

Figure 2 represents the prevalence of the four most frequent health conditions in included countries by working hours per week. The prevalence of each health condition is shown as a percentage. Figure 2 is divided into four parts by the following health conditions: 

**A:***Hypertension.* Our results show that hypertension occurs more frequently (more than 30%) according to the number of hours worked (more than 40 working hours per week) in the following countries: Hungary, Czech Republic, and Portugal. In Estonia, Sweden, and Germany, there was also a high frequency of hypertension, but participants reported working less than 40 h per week on average.

**B:***Heart Attack.* The prevalence of heart attack was the most frequent in Estonia (more than 10%), where participants work a little less than 40 h per week. In countries where participants were working more than 40 h per week (Hungary, Czech Republic, Portugal, Slovenia, and Poland), the prevalence of heart attack was between 2.5% and 10%.

**C:***Cholesterol.* Results show that cholesterol was the most frequently reported by participants in Portugal (more than 30%), where participants worked more than 40 h per week. Self-reported cholesterol ranged between 15% and 20% in Eastern European countries (Poland, Slovenia, Czech Republic, and Hungary), where participants worked more than 40 h per week. Prevalence of cholesterol was lower (between 10% and 15%) in the following countries: Netherlands, Sweden, Italy, and Switzerland. In these countries, participants were working less than 40 h per week.

**D:** *Diabetes*. Diabetes occurs the most frequently (more than 7.5%) in countries where participants worked more than 40 h per week (Portugal, Hungary, Poland, and the Czech Republic). The prevalence of diabetes was lower (less than 5%) in Denmark and Switzerland—participants worked less than 40 h per week.

### 3.3. Longitudinal Analysis of Health Outcomes between Healthcare Workers and Workers in Other Industries

**Figure 3:**
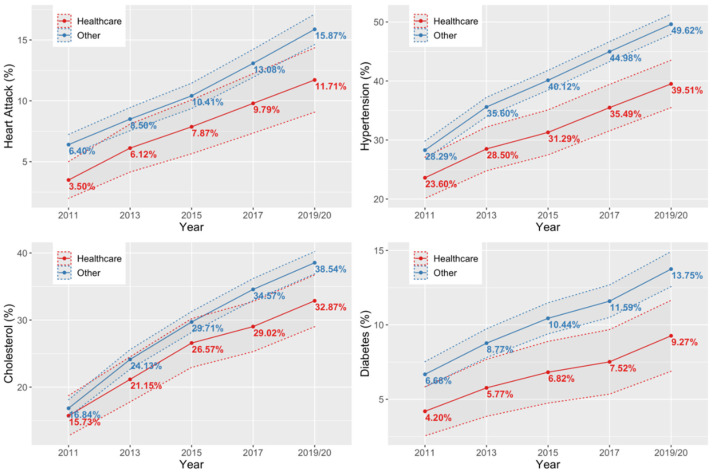
Longitudinal comparison of health condition prevalence between healthcare workers and other industries.

Figure 3 represents health outcomes in percentage among healthcare workers compared with other industries for the survey waves conducted in 2011, 2013, 2015, 2017, and 2019/2020. Only participants who answered the questions on their health conditions in all study waves from 2011 to 2019/2020 were included in the analysis (n = 3867). Among them, 572 (14.79%) were employees in the health and social sector. Figure 3 shows prevalence data for the four most prevalent health conditions. As we observe the data longitudinally for the same patient throughout the observation period, the prevalence steadily increases. In 2011, the prevalence of heart attack among healthcare workers was 3.50%. In comparison to other industries, there were 6.49% of participants with a heart attack. Over the years, the prevalence of heart attack raised to 11.71% among healthcare workers and 15.87% in other industries in the year 2019/2020. The prevalence of hypertension also increases among healthcare workers and other industries over the years. Among healthcare workers, the prevalence of hypertension raised by 15.91% from 2011 to 2019/2020; meanwhile, in other industries, the prevalence of hypertension raised by 21.33%. The largest increase in prevalence was reported for cholesterol, which occurred in 15.73% of healthcare workers and 16.84% in other industries in 2011. Over the observed period, the prevalence of cholesterol raised to 32.67% among healthcare workers and to 38.54% in other industries. The last health condition that was involved in the research is diabetes. The prevalence of diabetes also raised from 2011 to 2019/2020. Among healthcare workers, diabetes raised by 5.07% in the observed period. In other industries, the prevalence of diabetes increased by 7.07% in the observed nine-year period.

## 4. Discussion

This study aimed to determine the relationships between working long hours per week with health outcomes in different European countries by analyzing data from the SHARE survey database. 

We have found that, in every country covered by the survey, men are working longer hours per week in comparison to women. Similar results were also obtained in a European study in 2016, where they found that, in most countries, working moderately long hours was more frequent among men [17]. Data show that cardiovascular diseases cause more than 3.8 million deaths across Europe annually, about 1.76 million deaths in men and more than two million in women [22]. We also found that employees in Eastern Europe work more hours compared with Northern, Southern, and Western Europe. In those countries, hypertension, heart attack, cholesterol, and diabetes occur more often than in other European countries. From 1990 to 2015, an increase in the prevalence of cardiovascular diseases was reported in most European countries, which is most likely to be due to an aging population and population growth [22]. Similar to what the authors Dayoub & Jena [8] note, we found that the prevalence of heart attack, cholesterol, hypertension, and diabetes is lower among healthcare workers compared with employees in other industries but is still growing. The greatest increase is in the prevalence of cholesterol, both among healthcare workers and other employees, which may be the result of psychological stress [23].

*Association of Reported Health Conditions and Working Hours by Country Groups*

As found in a previous study [24], there is a strong connection between working long hours and reported health outcomes, which is in line with the belief that working more hours per week relates to health outcomes. The results show that health outcome is not always associated with working long hours. Hypertension is very frequent (more than 35%) in Germany, where participants work less than 36 h per week. The most important behavioral risk factors for cardiovascular disease are unhealthy diet, physical inactivity, smoking tobacco, and excessive alcohol consumption. Those risk factors are often shown as raised blood pressure, raised blood glucose, raised blood lipids, and overweight and obesity [25], which is still the major risk factor [26]. Especially in Eastern Europe, participants work 40 or more hours per week, except in Estonia, where women work slightly less than 40 h per week. The prevalence of hypertension is high (more than 30%), except in Slovenia and Poland, where hypertension occurs in slightly less than 25% of cases. Chronic diseases have reached epidemic proportions affecting 50% of the world’s population and consuming 86% of healthcare expenditure [27]. Therefore, prevention is important, and countries have different policies. Slovenia participated in the movement to share more data with the population about hypertension with the slogan: “Know your numbers—Know your blood pressure.” [28] and is still implementing the program “Together for Health”, in which experts provide support for a healthier lifestyle [29]. Moreover, since 2011, Slovenia has been introducing a concept of an advanced family medicine practice that aims to enable more efficient and comprehensive treatment of adult patients. In those family medicine centers, a nurse actively takes care of the preventive examinations of the defined target population (people over 30 years old) and holistic care of patients with chronic noncommunicable diseases [30]. In Poland, researchers created and tested a new model to provide professional monitoring and counseling on blood pressure by community pharmacies [31]. Therefore, the differences in the countries can be the consequences of different national strategies, such as preventive strategies and screenings.

In Northern Europe, all participants work 40 or fewer hours per week. The prevalence of chronic diseases is higher in Sweden (hypertension more than 30%, heart attack about 8%, and diabetes about 7.5%). In the Netherlands and Denmark, the prevalence of heart attack, hypertension, and diabetes is lower than in Sweden, except for cholesterol, which, in all countries in Northern Europe, occurs at about 15%. A similar increase in heart diseases in Northern Europe is cited by Dalstra et al. [32], especially among the working population. This may be linked to the fact that people in Southern Europe often eat the Mediterranean diet, which is clearly and strongly associated with better cardiovascular health outcomes [33]. Moreover, Southern Europe lagged in the spread of smoking prevalence compared with Northern Europe [34], which could also be correlated. On the other hand, in Southern Europe, men work more than 40 h per week and women work 40 or fewer hours per week. The higher prevalence of hypertension, heart attack, cholesterol, and diabetes is in Portugal (hypertension > 30%, heart attack > 5%, cholesterol > 30%, and diabetes > 10%). Moreover, the prevalence of diabetes and cholesterol is the highest compared with all European countries. Stroke and heart diseases were one of the leading causes of death in 2014. This may be associated with the fact that about one of six (16.1%) adults in Portugal is dealing with obesity, which is nearly one percentage point above the European average, especially given that poor diet and lack of physical activity can lead to high blood pressure, high cholesterol, diabetes, and other risk factors for cardiovascular diseases [34].

*Health Outcomes of Healthcare Workers in Comparison to Other Industries*

The results of our study show a marked increase in the prevalence of all observed health conditions over all industries—especially cholesterol, followed by hypertension. We cannot a priori identify which jobs can be considered most subjected to psycho-social risk [35] but we showed a slower increase in health conditions among healthcare workers compared with other industries. A Brazilian study found a high incidence of hypertension in 864 employees in university general hospital but mainly in those who were not physicians or members of the nursing staff [36]. First, this can be attributed to the fact that rates of obesity are lower among healthcare workers compared with the general population; also, there was better physical activity among healthcare workers and a lower prevalence of smoking [8]. Secondly, the level of digital health literacy among healthcare workers is assessed as desirable, which means that healthcare workers have an appropriate ability to help patients in self-care [37] and use that knowledge to improve their own healthy lifestyles. In all industries, the increase in cholesterol, hypertension, heart attack, and diabetes from 2011 to 2019/2020 is enormous. The incidence of cardiovascular diseases is also worrying in other non-European countries. For example, in Saudi Arabia, they have found that 10% of employees and students have elevated blood sugar and 17.5% of them have elevated cholesterol levels [38]. In Mississippi, they found 31.4% workers with hypertension in a three-year study that involved 1.1 million participants [39]. Results of a survey in Namibia showed high blood glucose and diabetes were the biggest contributors to absenteeism among employees in seven industries [40]. The crisis with chronic diseases (cardiovascular diseases, diabetes, pulmonary diseases, and several forms of cancer) has to be addressed because they are the leading causes of morbidity and mortality globally. With improvements in physical activity, nutrition, and tobacco use, there is hope for improving the health status of employees worldwide [41].

*Limitations*

Data on the health status of the interviewees collected in the SHARE database are based on the self-assessment of the interviewees and not based on objective measurements of their health condition. Moreover, participants working in five industries (mining, electricity, hotels, financial, and real estate), where the sample was not representative, were excluded from the analysis.

## 5. Conclusions

In conclusion, the increase in the prevalence of self-perceived health conditions (cholesterol, hypertension, diabetes, and heart attack) from 2011 to 2019/2020 among employees in European countries is enormous. The prevalence of cholesterol raised for 17.14% among healthcare workers and 21.70% in other industries over the observed nine-year period, which is the largest increase of all observed health conditions. Cholesterol is closely related to lifestyle and working stressors; that is why it is essential for employees to have a good work–life balance. Prevention is most important; however, it should be noted that there is a high variance in policies among different countries. Each country should participate in assessing the workload, risk factors, and stressors in every industry, as the incidence of work-related diseases varies depending on the industry. Managers in companies should create a multi-professional, transdisciplinary team to design working environment, which would encourage a healthier lifestyle among employees. It is necessary to adapt the workplace and tasks to their specific needs in every industry to minimize risks. Additional research is needed in areas where the sample was not representative.

## Data Availability

The data were obtained from the Survey of Health, Ageing and Retirement in Europe (SHARE) database: http://www.share-project.org/data-access/user-registration.html?L= (accessed on 15 December 2021).

## References

[1] Wolf J., Prüss-Ustün A., Ivanov I., Mugdal S., Corvalán C., Bos R., Neira M., World Health Organisation (2018). Preventing Disease through a Healthier and Safer Workplace.

[2] European Agency for Safety and Health at Work (EU-OSHA) (2019). The Value of Occupational Safety and Health and the Societal Costs of Work-Related Injuries and Diseases: European Risk Observatory, Literature Review.

[3] European Agency for Safety and Health at Work (EU-OSHA) (2017). Estimating the Costs of Work-Related Accidents and Ill-Health: An analysis of European Data Sources.

[4] Dutch National Institute for Public Health and the Environment (2012). Europeans of Retirement Age: Chronic Diseases and Economic Activity.

[5] van der Heide I., Snoeijs S., Melchiorre M.G., Quattrini S., Boerma W., Schellevis F., Rijken M. (2015). Innovating Care for People with Multiple Chronic Conditions in Europe.

[6] Steptoe A., Kivimäki M. (2012). Stress and cardiovascular disease. Nat. Rev. Cardiol..

[7] Nielsen K., Jørgensen M.B., Milczarek M., Munar L. (2018). Healthy Workers, Thriving Companies—A Practical Guide to Wellbeing at Work: Tackling Psychosocial Risks and Musculoskeletal Disorders in Small Businesses.

[8] Dayoub E., Jena A.B. (2015). Chronic Disease Prevalence and Healthy Lifestyle Behaviors Among, U.S. Health Care Professionals. Mayo Clin. Proc..

[9] Rössler W. (2012). Stress, burnout, and job dissatisfaction in mental health workers. Eur. Arch. Psychiatry Clin. Neurosci..

[10] International Labour Organisation (2015). Improving Employment and Working Conditions in Health Services.

[11] Griffiths P., Dall’Ora C., Simon M., Ball J., Lindqvist R., Rafferty A.M., Schoonhoven L., Tishelman C., Aiken L.H. (2014). RN4CAST Consortium. Nurses’ Shift Length and Overtime Working in 12 European Countries. Med. Care.

[12] (2003). European Parliament, Council of the European Union. https://eurlex.europa.eu/legalcontent/EN/ALL/?uri=CELEX:32003L0088.

[13] World Health Organisation (2021). https://www.who.int/news-room/questions-and-answers/item/global-regional-and-national-burdens-of-ischemic-heart-disease-and-stroke-attributable-to-exposure-to-long-working-hours-for-194-countries-2000-2016.

[14] (2022). Eurostat Statistics. https://ec.europa.eu/eurostat/statistics-explained/index.php?title=Hours_of_work_-_annual_statistics.

[15] Piasna A. (2018). Scheduled to work hard: The relationship between non-standard working hours and work intensity among European workers [2005–2015]. Hum. Resour. Manag. J..

[16] Artazcoz L., Cortès I., Escribà-Agüir V., Bartoll X., Basart H., Borrell C. (2013). Long working hours and health status among employees in Europe. Scand. J. Work Environ. Health.

[17] Artazcoz L., Cortès I., Benavides F.G., Escribà-Agüir V., Bartoll X., Vargas H., Borrell C. (2016). Long working hours and health in Europe: Gender and welfare state differences in a context of economic crisis. Health Place.

[18] (2021). Survey of Health. Ageing and Retirement in Europe. SHARE-Survey of Health, Ageing and Retirement in Europe. http://www.share-project.org/home0.html.

[19] Börsch-Supan A., Brandt M., Hunkler C., Kneip T., Korbmacher J., Malter F., Schaan B., Stuck S., Zuber S. (2013). Data Resource Profile: The Survey of Health, Ageing and Retirement in Europe [SHARE]. Int. J. Epidemiol..

[20] R Development Core Team (2005). A Language and Environment for Statistical Computing.

[21] Štiglic G., Roger W., Cilar L. (2019). R you ready? Using the R programme for statistical analysis and graphics. Res. Nurs. Health.

[22] Townsend N., Kazakiewicz D., Wright F.L., Timms A., Huculeci R., Torbica A., Gale C.P., Achenbach S., Weidinger F., Vardas P. (2022). Epidemiology of cardiovascular disease in Europe. Nat. Rev. Cardiol..

[23] Assadi S.N. (2017). What are the effects of psychological stress and physical work on blood lipid profiles?. Medicine.

[24] Gangster D.C., Rosen C.C., Fisher G.G. (2016). Long Working Hours and Well-being: What We Know, What We Do Not Know, and What We Need to Know. J. Bus. Psychol..

[25] World Health Organisation (2021). Cardiovascular Diseases [CVDs]. https://www.who.int/news-room/fact-sheets/detail/cardiovascular-diseases-[cvds].

[26] Kivimäki M., Kuosma E., Ferrie J.E., Luukkonen R., Nyberg S.T., Alfredsson L., Batty G.D., Brunner E.J., Fransson E., Goldberg M. (2017). Overweight, obesity, and risk of cardiometabolic multimorbidity: Pooled analysis of individual-level data for 120 813 adults from 16 cohort studies from the USA and Europe. Lancet Public Health.

[27] Holman H.R. (2020). The Relation of the Chronic Disease Epidemic to the Health Care Crisis. ACR Open Rheumatol..

[28] Nacionalni inštitut za javno zdravje (2017). Bolezni. https://www.nijz.si/sl/17-maj-svetovni-dan-hipertenzije-2017.

[29] Nacionalni inštitut za javno zdravje (2020). Program Skupaj za Zdravje. https://www.nijz.si/sl/program-skupaj-za-zdravje.

[30] Ministrstvo za zdravje (2019). Učinkovitost Dela Ambulant Družinske Medicine za Področje Nalog Diplomirane Medicinske Sestre.

[31] Waszyk-Nowaczyk M., Guzenda W., Plewka B., Michalak M., Cerbin-Koczorowska M., Stryczyński Ł., Byliniak M., Ratka A. (2020). Screening Services in a Community Pharmacy in Poznan [Poland] to Increase Early Detection of Hypertension. J. Clin. Med..

[32] Dalstra J.A., Kunst A.E., Borrell C., Breeze E., Cambois E., Costa G., Geurts J.J.M., Lahelma E., Van Oyen H., Rasmussen N.K. (2005). Socioeconomic differences in the prevalence of common chronic diseases: An overview of eight European countries. Int. J. Epidemiol..

[33] Martínez-González M.A., Gea A., Ruiz-Canela M. (2019). The Mediterranean Diet and Cardiovascular Health. Circ. Res..

[34] OECD/European Observatory on Health Systems and Policies (2017). Portugal: Country Health Profile 2017.

[35] Eurofound (2012). Trends in Job Quality in Europe.

[36] Mion Jr D., Pierin A.M., Bambirra A.P., Assunção J.H., Monteiro J.M., Chinen R.Y., Coser R.B., Aikawa V.N., Cação F.M., Hausen M. (2004). Hypertension in employees of a University General Hospital. Rev. Do Hosp. Das Clínicas.

[37] Alipour J., Payandeh A. (2022). Assessing the level of digital health literacy among healthcare workers of teaching hospitals in the southeast of Iran. Inform. Med. Unlocked.

[38] Altemani A. (2016). Prevalence of diabetes mellitus, hypertension and hyperlipidemia among students and employees in University of Tabuk, Saudi Arabia. Eur. Sci. J..

[39] Mendy V.L., Vargas R., Ogungbe O., Zhang L. (2020). Hypertension among Mississippi workers by sociodemographic characteristics and occupation, behavioral risk factor surveillance system. Int. J. Hyperten.

[40] Guariguata L., de Beer I., Hough R., Bindels E., Weimers-Maasdorp D., Feeley F.G., Rinke de Wit T.F. (2012). Diabetes, HIV and other health determinants associated with absenteeism among formal sector workers in Namibia. BMC Public Health.

[41] Arena R., McNeil A., Sagner M., Hills A.P. (2017). The Current Global State of Key Lifestyle Characteristics: Health and Economic Implications. Prog. Cardiovasc. Dis..

